# Evaluation of thermocycling, color stability, surface roughness and hardness of nanocarbon HPP, BioHPP and PEEK used for fabrication of prosthetic restorations ( in vitro study)

**DOI:** 10.1186/s12903-026-09615-6

**Published:** 2026-08-31

**Authors:** Mohamed Z. Basiony, Rim A. Selima, Nermeen A. Rady, Sherif M. Abdel Hamid

**Affiliations:** 1 Prosthodontics Department, Faculty of Dentistry, Pharos University in Alexandria, Alexandria, Egypt; 2 Prosthodontics Department, Faculty of Dentistry, Alexandria University, Alexandria, Egypt

**Keywords:** BioHPP, PEEK, Nano‑carbon-HPP, Implant overdenture, Surface roughness, Hardness, Color stability

## Abstract

**Statement of problem:**

The selection of appropriate dental materials plays a crucial role in the success of prosthetic restorations. With the limitations of metallic alloys, including their aesthetic shortcomings, weight, corrosion potential, and cost, there is growing interest in high-performance polymer alternatives.

**Purpose:**

The objective of the present study was to evaluate color stability, surface roughness and Hardness of BioHPP, Nanocarbon HPP and PEEK used for fabrication of prosthetic restorations (An In Vitro Study).

**Material and methods:**

A total of 42 cylindrical samples (14 per group) were divided into three groups: Group 1: nanocarbon High-Performance Polymer, Group 2: BioHPP, Group 3: PEEK. They were fabricated using CAD/CAM technology and subjected to thermocyclic aging. Color stability was assessed spectrophotometrically after immersion in a staining medium over 28 days. Surface roughness was measured using a profilometer, while hardness was evaluated using the Vickers hardness test.

**Results:**

Statistically significant differences were observed among all three materials in color stability, surface roughness, and hardness (*p* < 0.001). PEEK demonstrated the highest surface roughness, while nano-carbon reinforced polymersexhibited the lowest surface roughness and highest hardness values. In terms of color stability, all materials showed varying degrees of discoloration over time, but nano-carbon-HPP generally maintained better stability.

**Conclusion:**

Nano-carbon-HPP demonstrated superior mechanical performance, combining low surface roughness with high hardness, making them a promising alternative to traditional materials for full-arch dental prostheses. These findings highlight the potential of nano-carbon materials to improve durability, patient comfort, and aesthetic outcomes in prosthodontic applications.

**Clinical implication:**

The use of nano‑carbon reinforced polymers may improve the longevity, esthetics, and patient comfort of implant‑supported overdentures.

## Introduction

While the prosthetic rehabilitation of total edentulism remains a cornerstone of restorative practice, managing the severely atrophic mandible presents a formidable clinical hurdle. This complexity is frequently exacerbated in the geriatric population, where advanced ridge resorption intersects with a diminished neuromuscular capacity to adapt to new prostheses [1]. 

Integrating dental implants into the overdenture design addresses the primary shortcomings of conventional removable prosthodontics. This modality effectively arrests mandibular bone loss and optimizes bolus processing during mastication, translating to superior patient-reported outcomes and a higher degree of physiological well-being [2]. 

Material selection is a critical determinant of long-term success, as the inherent mechanical properties of the restorative medium govern the transmission of occlusal forces to the peri-implant architecture. Specifically, the elastic modulus of the material dictates the stress distribution patterns within the implant-bone interface; contrary to some intuitive assumptions, current literature suggests that more compliant (less rigid) materials may precipitate higher stress concentrations in prosthetic components and the underlying bone complex due to increased flexural deformation.

The integration of metallic frameworks in implant prosthodontics serves as a strategic reinforcement to optimize the assembly’s flexural rigidity. By establishing a rigid splinting effect across the implants, these frameworks mitigate the risk of catastrophic material failure while facilitating a more homogenous distribution of occlusal loads. Prevailing biomechanical theory suggests that high-modulus materials minimize localized strain at the abutment-implant interface, thereby safeguarding the structural integrity of the prosthetic complex [3].

While metallic reinforcements provide high rigidity, they are increasingly scrutinized for their lack of adaptability and poor esthetic integration. The high density of these materials translates into a heavy prosthetic profile that can diminish the wearer’s experience, particularly in extensive FP2 and FP3 reconstructions. Moreover, the risk of marginal mucosal discoloration and the significant fiscal overhead associated with alloy fabrication present ongoing challenges. These factors, alongside the difficulty of chairside adjustability, underscore the need for restorative materials that better balance biomechanical strength with biological and esthetic harmony [4].

The dental field is currently navigating a significant transition. For decades, metal alloys served as the undisputed benchmark against which all emerging materials were measured. However, the rapid maturation of CAD-CAM ecosystems has fundamentally altered this hierarchy. We are seeing a decisive move toward non-metallic prostheses, not merely for their aesthetics, but because digital manufacturing now allows us to harness the unique properties of polymers and ceramics with a precision that was previously unattainable.

In the search for alternatives to traditional titanium and zirconia, PEEK has emerged as a compelling candidate for diverse clinical applications. Its appeal lies primarily in its unique biomechanical profile; with an elastic modulus of approximately 3–4 GPa, PEEK effectively mimics the flexibility of human bone [5–8]. This ‘iso-elastic’ behavior provides a critical cushioning effect that attenuates the stress concentrations typically transferred to abutment teeth. Beyond its biomimetic flexibility, the material remains remarkably robust, exhibiting high fatigue and tensile strength alongside the thermal and electrical insulation properties necessary for a stable intraoral environment.

The recent introduction of BioHPP (Bio High-Performance Polymer) represents a significant milestone in the refinement of metal-free restorative options. By reinforcing a PEEK matrix with approximately 20% ceramic fillers, this material transcends the limitations of its unfilled counterparts. This ceramic integration not only augments the structural rigidity and mechanical resilience of the polymer but also optimizes its optical properties. Consequently, BioHPP is increasingly regarded as an indispensable addition to the prosthodontic toolkit, offering a balanced synergy between physiological compatibility and the aesthetic demands of contemporary implantology.

Carbon-based nano-allotropes have shown particular promise for reinforcing polymeric restorative materials [9]. Incorporating even a nominal concentration (1%) of carbon particles into a polymer matrix can significantly improve flexural and impact strength, although this gain in toughness is accompanied by a reduction in surface hardness, suggesting a more resilient, less brittle material profile [10, 11]. Compared with glass-fiber reinforcement, carbon-based reinforcement offers a superior biomechanical profile, combining high stiffness, fracture toughness, and resistance to abrasion and creep with low density, a negligible coefficient of thermal expansion, and chemical inertness, properties that together support its use in long-term intraoral applications.

Therefore, the aim of the present study was to evaluate color stability, surface roughness and Hardness of BioHPP, Nanocarbon HPP and PEEK used for fabrication of prosthetic restoration.

The null hypothesis indicating that there is no statistically significant difference in the color stability, surface roughness, or hardness among BioHPP, PEEK, and nano-carbon reinforced dental materials.

## Materials and methods

Sample size was estimated based on assuming confidence level = 95% and study power = 80%. A comparison between the independent means of BioHPP, PEEK and nano-carbon indicated that a sample size of 14 samples per group was required. Total sample size = Number per group x Number of groups = 14 × 3 = 42 samples [5, 12, 13]. 

This was an in vitro experimental study in which the specimens were randomly allocated, using a computer-generated randomization sequence, into three groups according to the material used for their construction: group I PEEK, group II BioHPP, and group III involved nano-carbon reinforced polymers.

### Samples fabrication

**Fig. 1:**
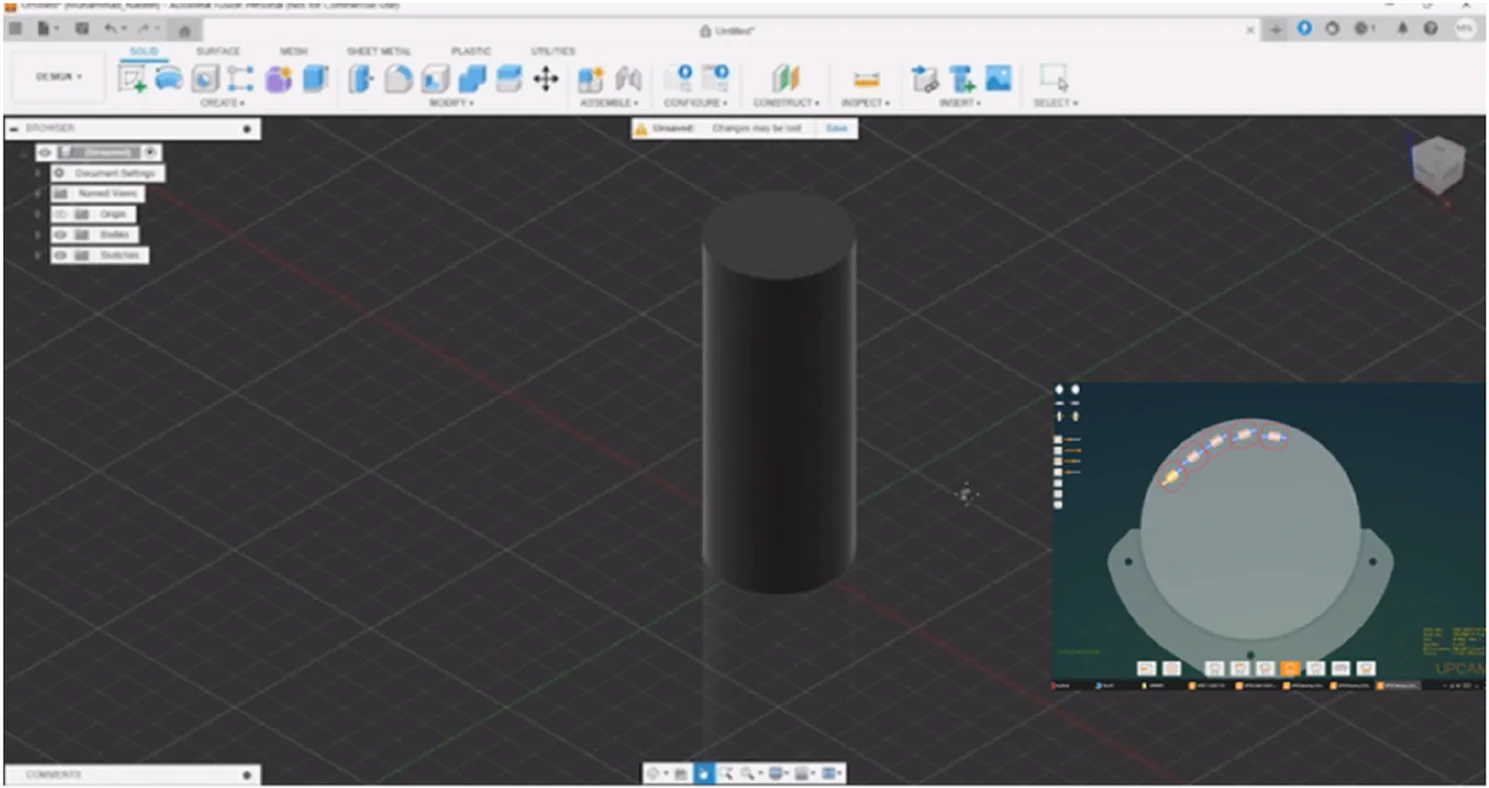
A cylindrical sample with a diameter of 2 mm and thickness of 5 mm were designed using EXOCAD software

Cylindrical samples with a diameter of 2 mm and thickness of 5 mm were designed using EXOCAD software and subsequently milled using CAD/CAM technology according to the manufacturer’s instructions, finished and finally polished with a low-speed handpiece and diamond polishing paste Renfert polish (Renfert, Hilzingen, Germany), were ultrasonically cleaned for 10 min, and were air-dried. color stability, surface roughness, hardness) were performed on the same in vitro specimen set (Fig. 1).

### Thermocyclic aging

**Fig. 2:**
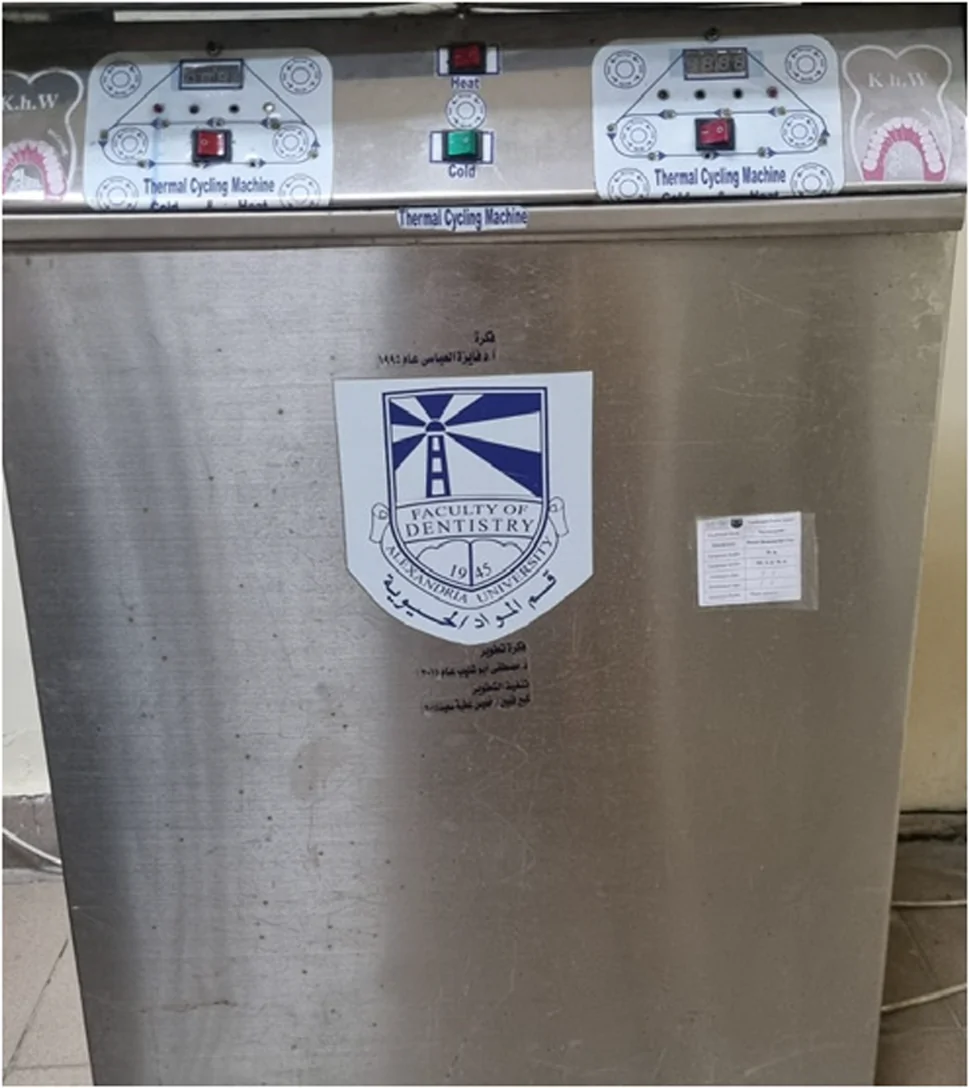
The specimens were then subjected to aging by thermal cycling in which A total of 10,000 cycles in the water bath was applied to simulate one year of oral use

The specimens were then subjected to aging by thermal cycling. A total of 10,000 cycles in the water bath (5–55◦C, 30 s dwell time and 10 s transfer time) was applied to simulate one year of oral use. This protocol was selected based on the standard ISO number 11,405 and simulates the temperature changes that take place in the oral environment [14] (Fig. 2).

### Color stability

#### Staining process

**Fig. 3:**
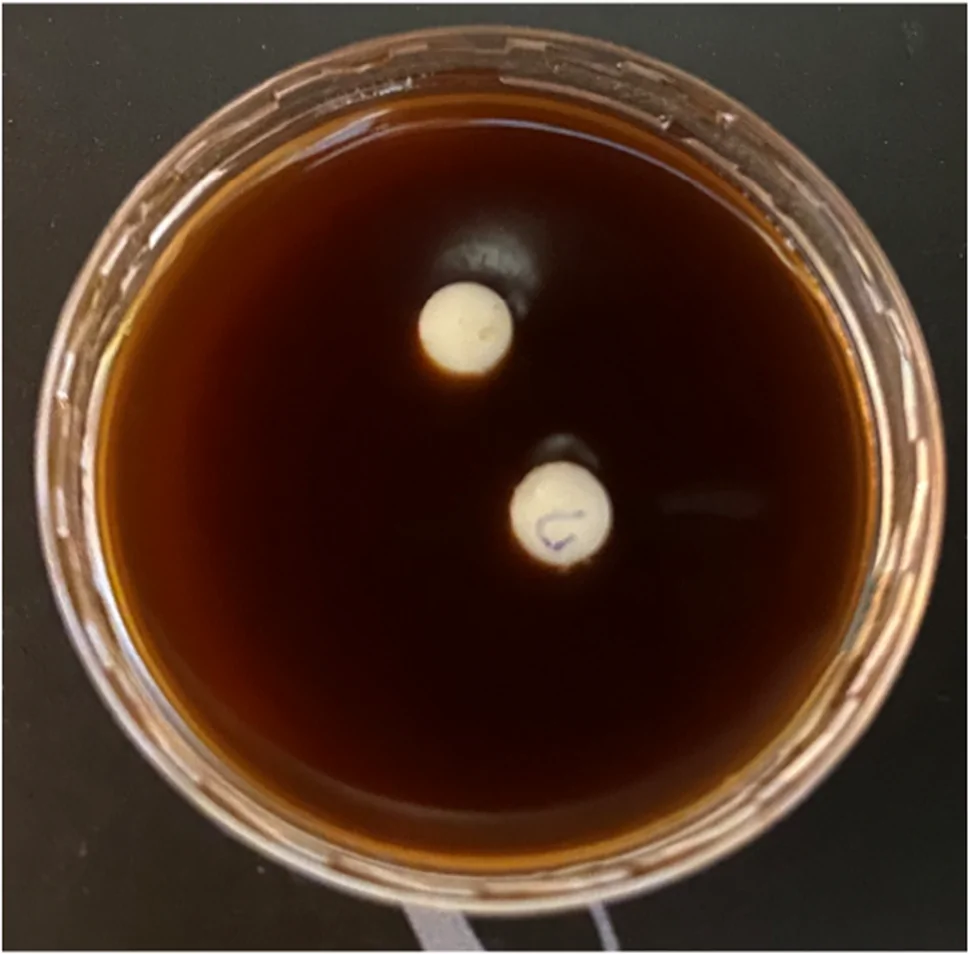
The specimens were immersed in staining solution at room temperature for 28-days test period

Staining process was carried out by using staining solution which was coffee (Nescafe Classic, Nestle, Vevey, Switzerland) as a staining agent. The specimens were immersed in staining solution at room temperature for 28-days test period. Solutions were changed daily and put in vials with cover that prevent evaporation of staining solutions. Spectrophotometric analysis was made immediately after light-polymerization (T0) and at 7 (T1), 14 (T2), 21 (T3), 28 (T4) days the beginning of the experimentation (Fig. 3).

**Fig. 4:**
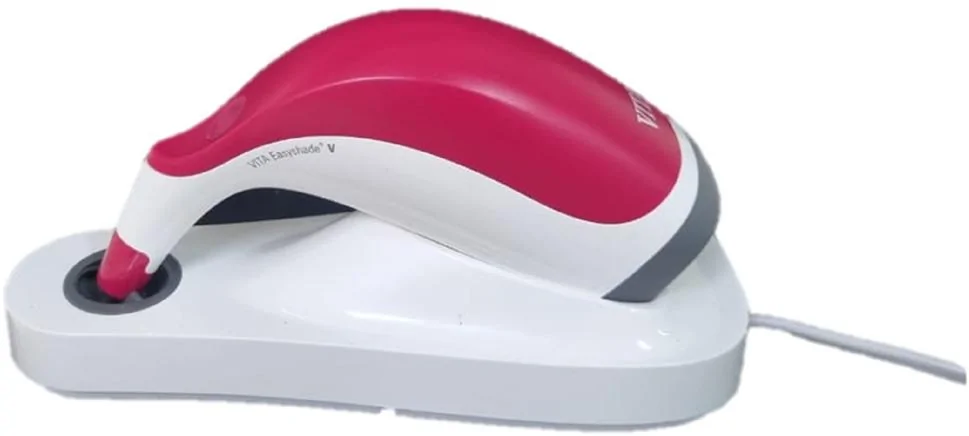
A digital spectrophotometer has been used to determine the precise and reliable shade matching for the specimens

A digital spectrophotometer (Vita Easyshade Compact, Vita Zahnfabrik, Bad Säckiningen, Germany) has been used to determine the precise and reliable shade matching for natural teeth and ceramic restorations. It was also used to evaluate the color stability of the crowns before and after thermocycling and the chewing simulator. The digital spectrophotrophotometer measured the wavelength range from 400 to 700 nm and used LED technology, which was unaffected by the environment (Fig. 4).

#### Spectrophotometer analysis

**Fig. 5:**
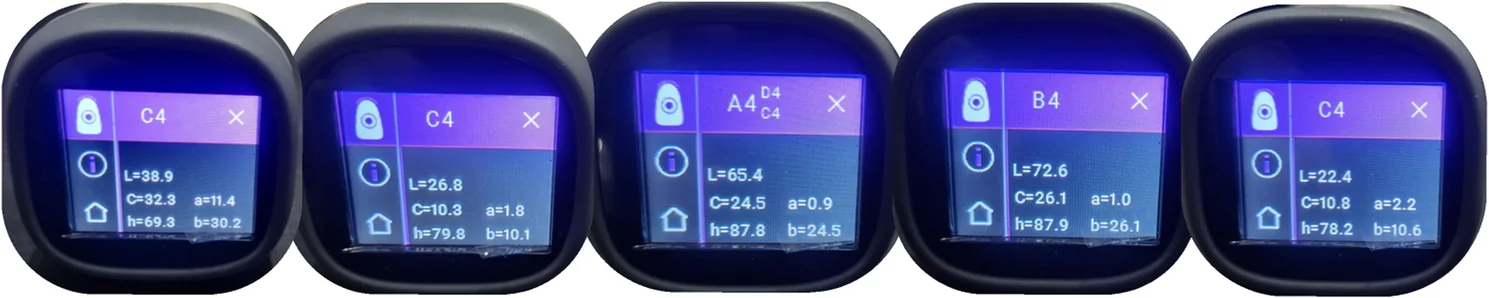
The reading of each sample was recorded for future analysis

Spectrophotometric analysis was made to determine the precise and reliable shade matching for the specimens, immediately after light-polymerization at the beginning of the study and at 7, 14, 21, 28 days. It was also used to evaluate the color stability of the specimens before and after thermocycling (Fig. 5).

Before each measurement, the specimens were gently rinsed with distilled water and air-dried. Color of the specimens was measured with a spectrophotometer against a black background time, late the absence of light in the mouth and against a white background. All specimens were chromatically measured 4 times, and the average values were calculated.

### Surface roughness

**Fig. 6:**
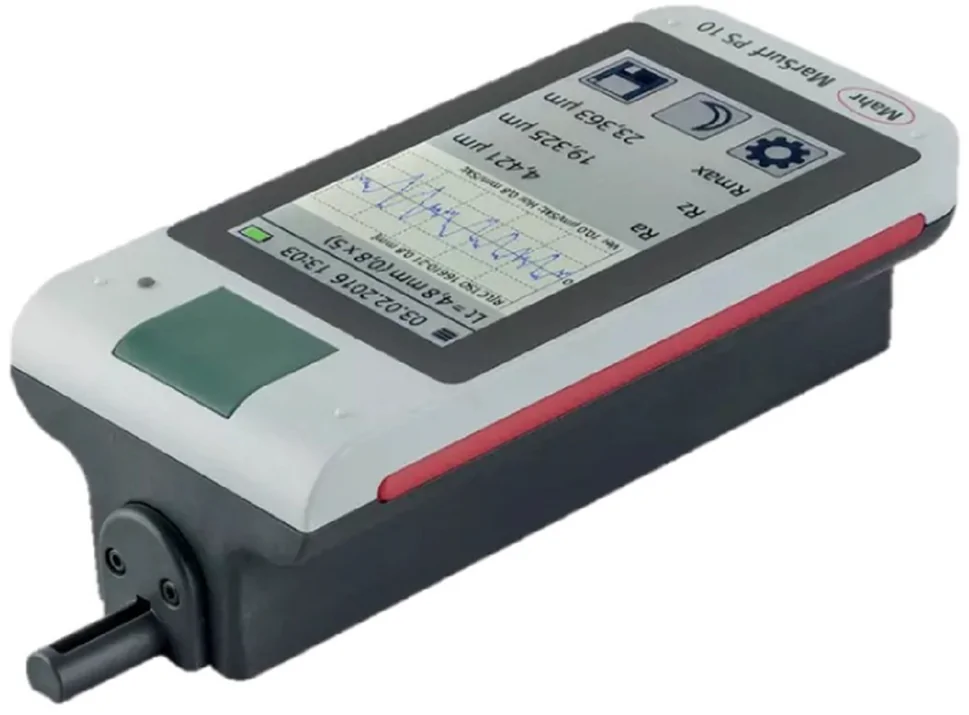
Surface roughness measurements were performed using a profilometer on all specimens from each group using 5 µm diamond stylus

Surface roughness (SR) measurements were performed using a profilometer (TR200, Beijing TIME High Technology, Beijing, China) on all specimens from each group (*n* = 14) using the 5 μm diamond stylus with 1.25 mm total length, 0.25 mm cut-off value, 0.02 μm resolution, and a Gaussian filter, according to ISO 4288:1996 [6] before and after each water immersion period and after aging, for each group (Fig. 6).

**Fig. 7:**
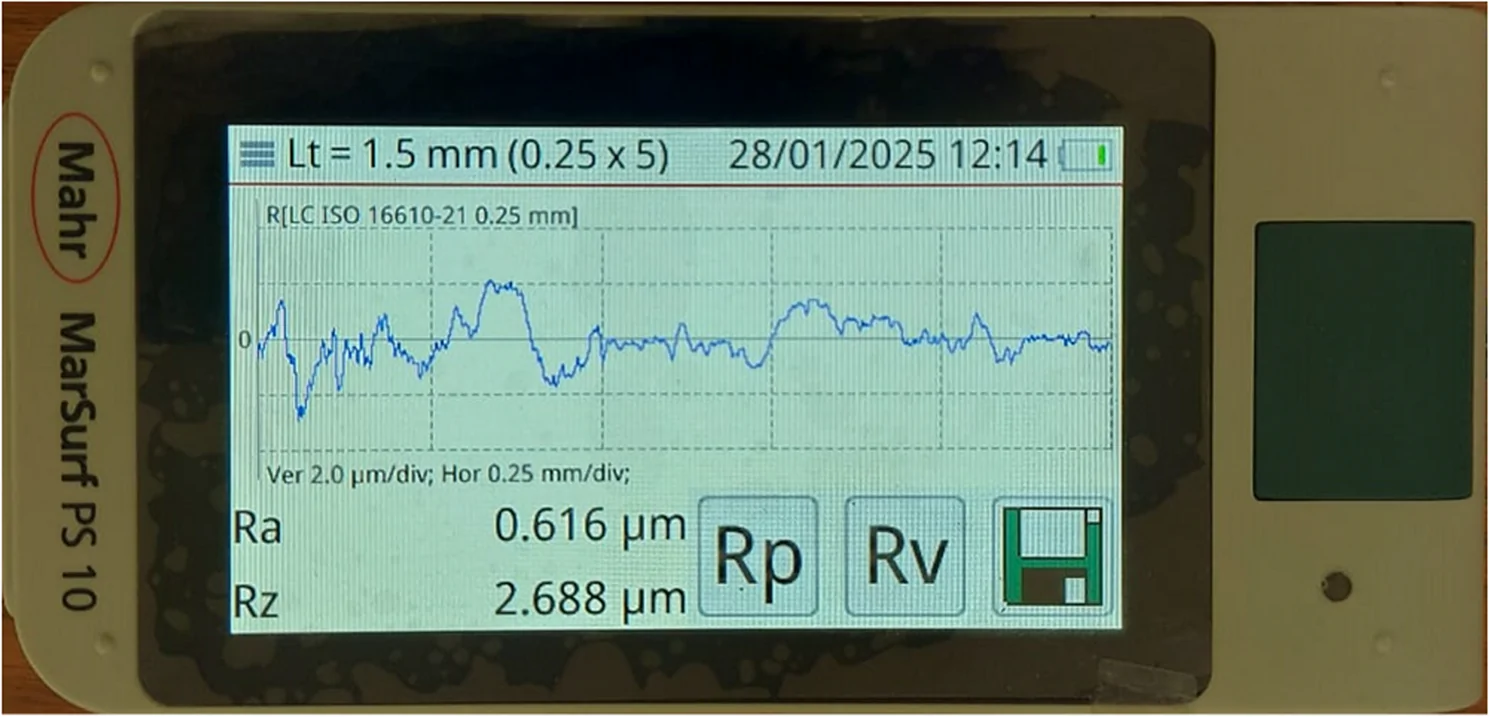
The arithmetic mean value of all peaks and valleys in the measured profile was analyzed

The arithmetic mean value of all peaks and valleys (Ra) in the measured profile was analyzed, and the average value from three measurements in three different directions was calculated for each specimen. The sampling length was 0.3 mm, and a force of 0.7 mN was applied (Fig. 7).

### Hardness

**Fig. 8:**
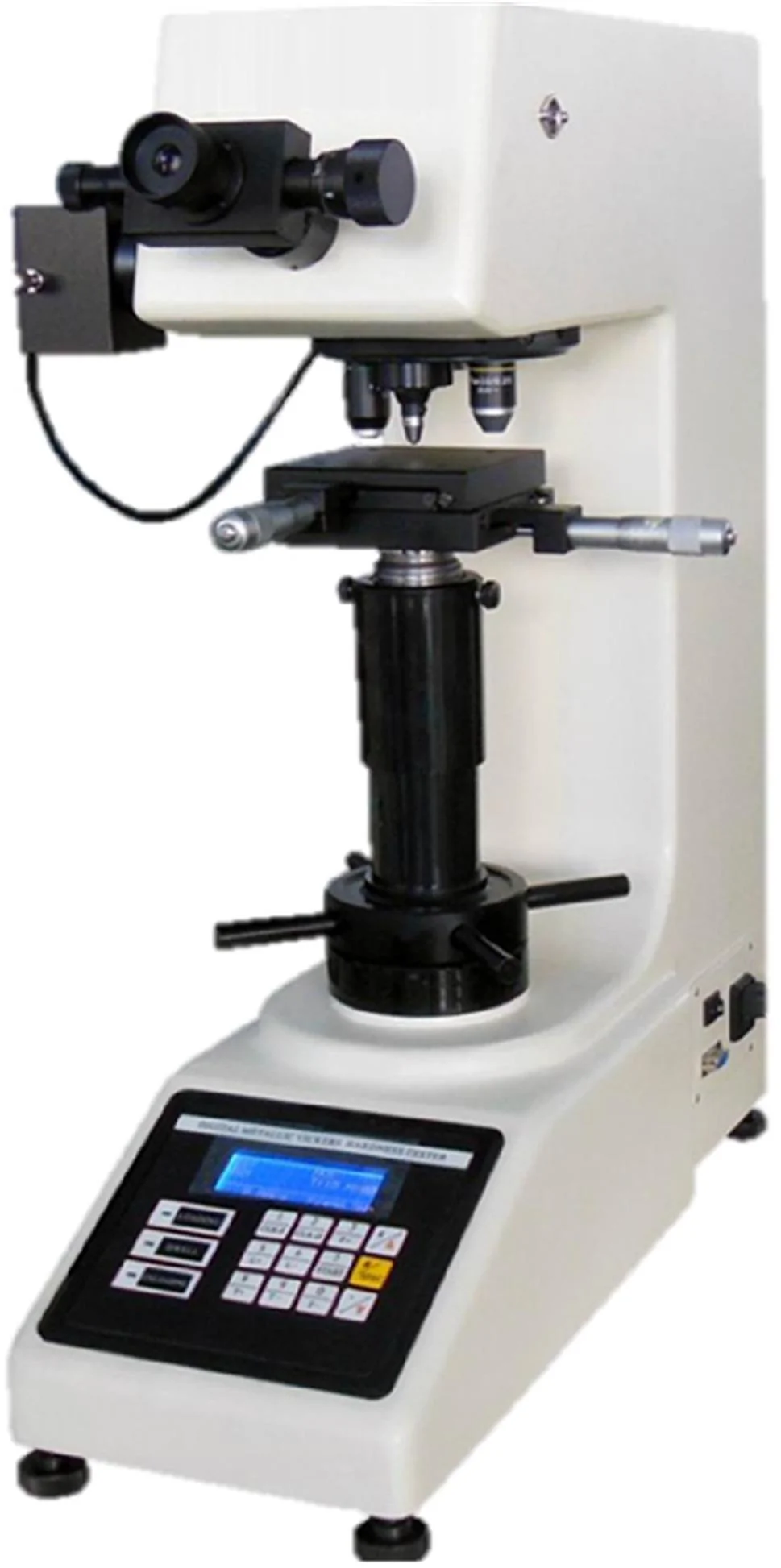
The Vickers hardness numbers for the tested samples were calculated using a Vickers micro-hardness tester under a 500 gf (4.9 N) load

**Fig. 9:**
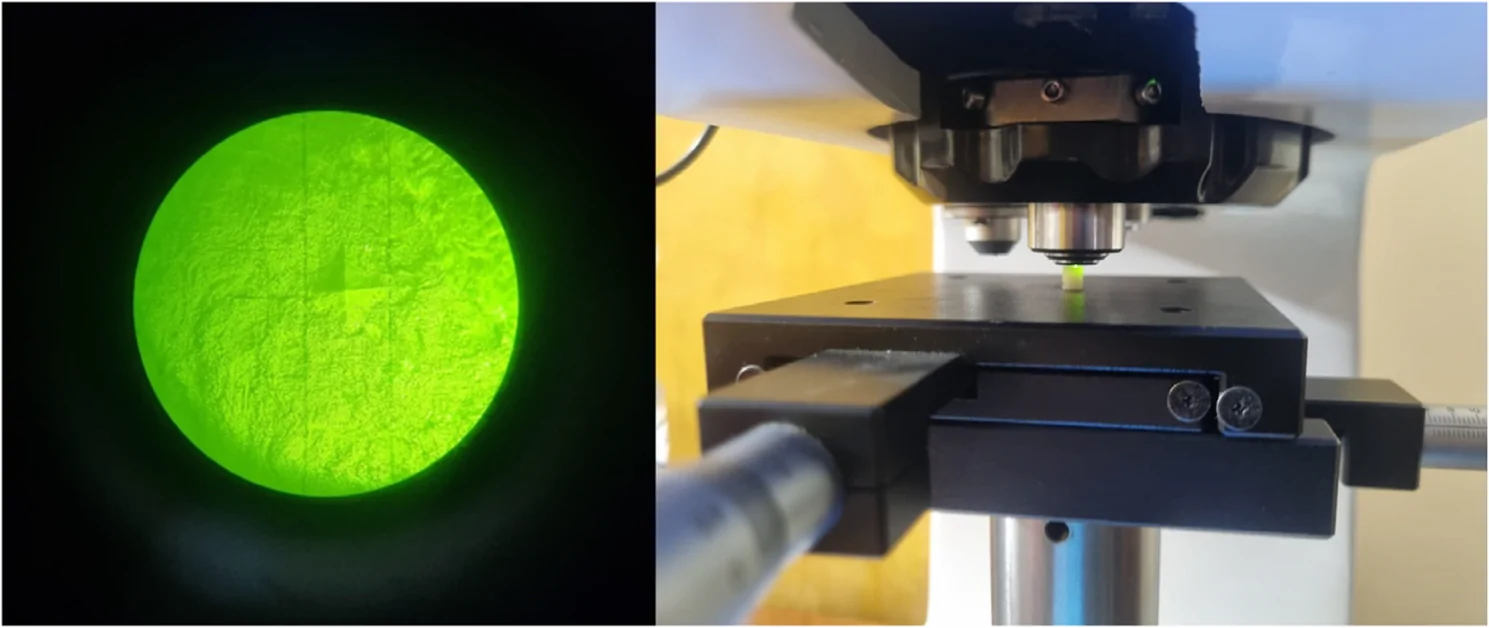
Five indentation readings were taken for each specimen, and the average of those readings was calculated

The Vickers hardness numbers (VHNs) for the tested samples were calculated using a Vickers microhardness tester (Fig. 8). A 500 gf (4.9 N) load was applied by the diamond indenter for a dwell time of 10 s. Five indentation readings were taken for each specimen, and the average of those readings was calculated. Then, for each group, surface hardness was assessed and statistically analyzed both before and after thermocycling to age the materials (Fig. 9).

## Results

### Statistical analysis

Data were analyzed using IBM SPSS (version 29.0) and R software (version 4.3.2). The achieved results undergo evaluation at a 5% Significance Level, and a 95% Confidence interval.

- ANOVA test was used to identify significance difference among the means of more than two groups, followed by a post hoc test to determine the difference between each pair.
- Nonparametric tests were conducted via Kruskal-Walli’s tests to detect significant differences in quantitative variables between different variables.
- Repeated ANOVA test was used to detect the significance of repeated measures of the same group. Graphics were used for data visualization.

### Evaluation of color stability

Table 1 this table provides descriptive statistics for each of the three material groups. BioHPP has the highest mean value, followed by Nano-carbon HPP and PEEK. 

**Table 1:**
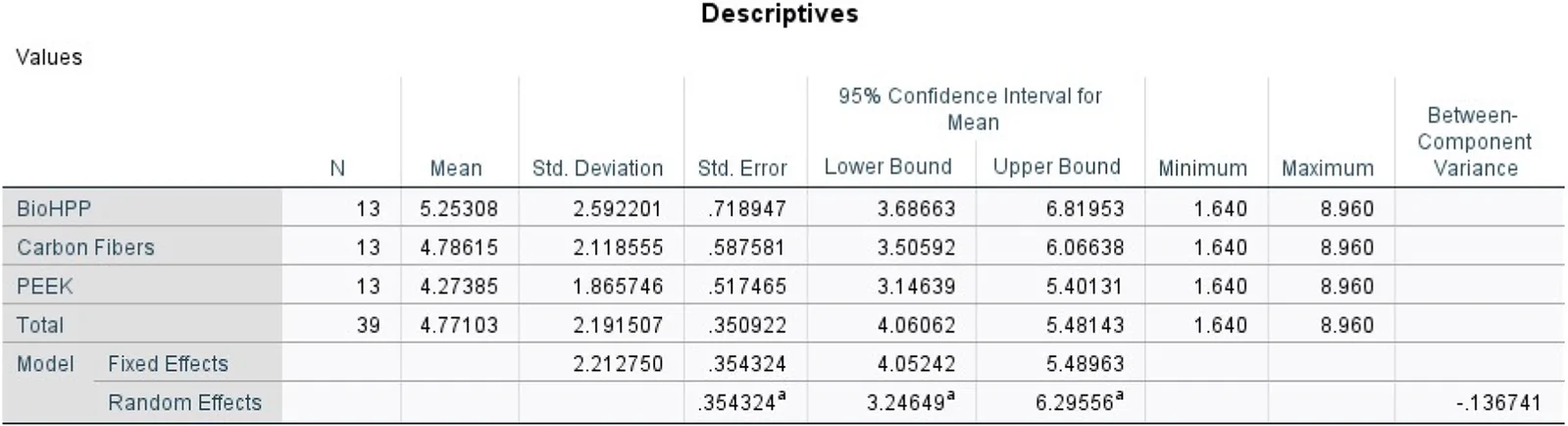
Descriptive statistics for each of the three material groups

### Evaluation of surface roughness

**Fig. 10:**
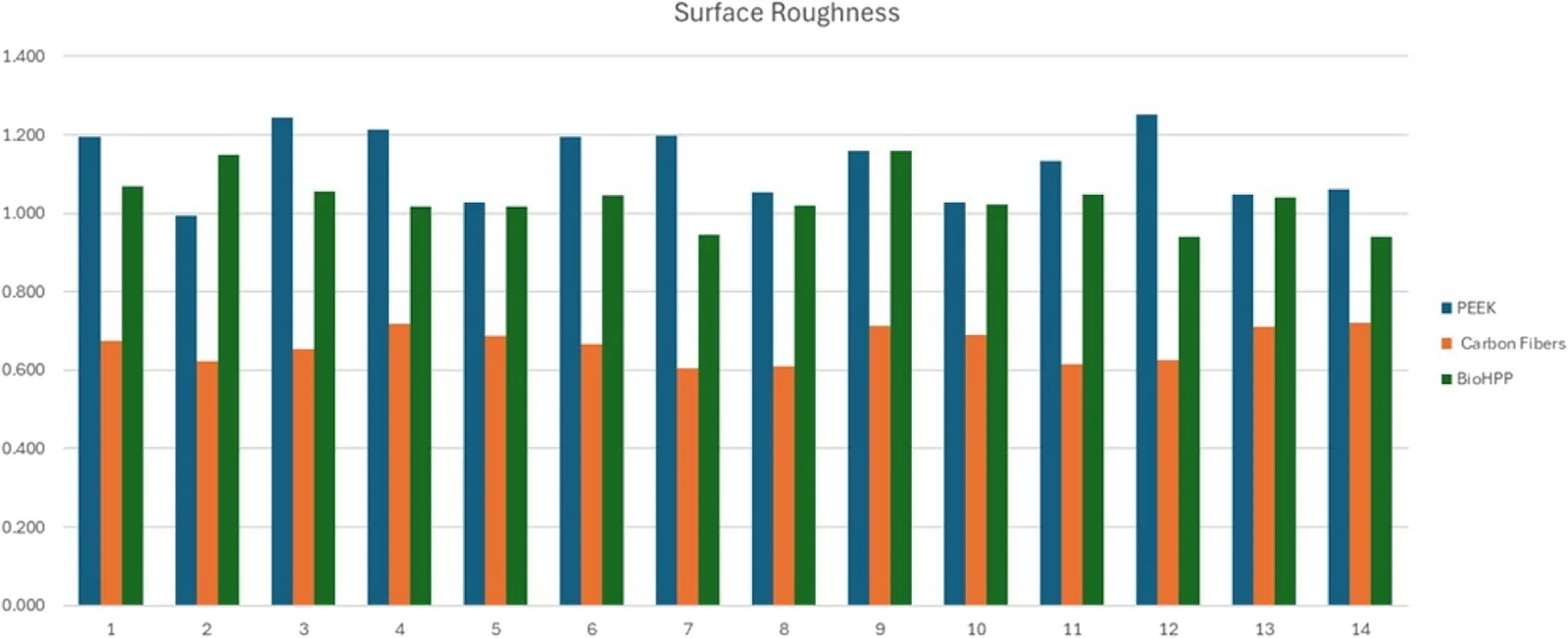
Compares the surface roughness of the same three materials. Nano-carbon HPP (orange bars) show the lowest surface roughness across all samples, whilw BioHPP shows higher roughness, often close to or just below PEEK. PEEK has the highest surface roughness in most samples

The tables presented summarize the results of a statistical comparison between three dental materials; group I BioHPP, group II Nano-carbon HPP, and group III PEEK, based on their measured values in an in vitro study (Tables 2, 3, 4 and Fig. 10).

**Table 2:**
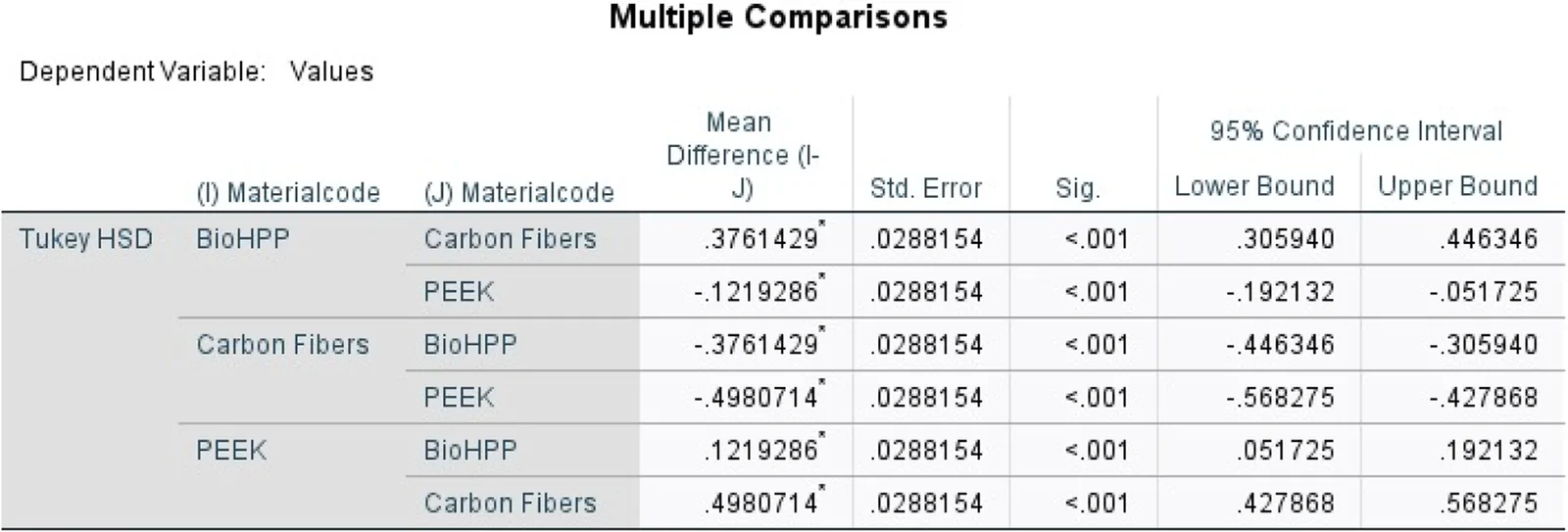
Shows the Tukey HSD post hoc test further confirms this, revealing that all pairwise comparisons between materials are statistically significant (*p* < 0.001). Specifically, PEEK showed significantly higher values than both BioHPP and Nano-carbon HPP, and BioHPP showed significantly higher values than Nano-carbon HPP

**Table 3:**
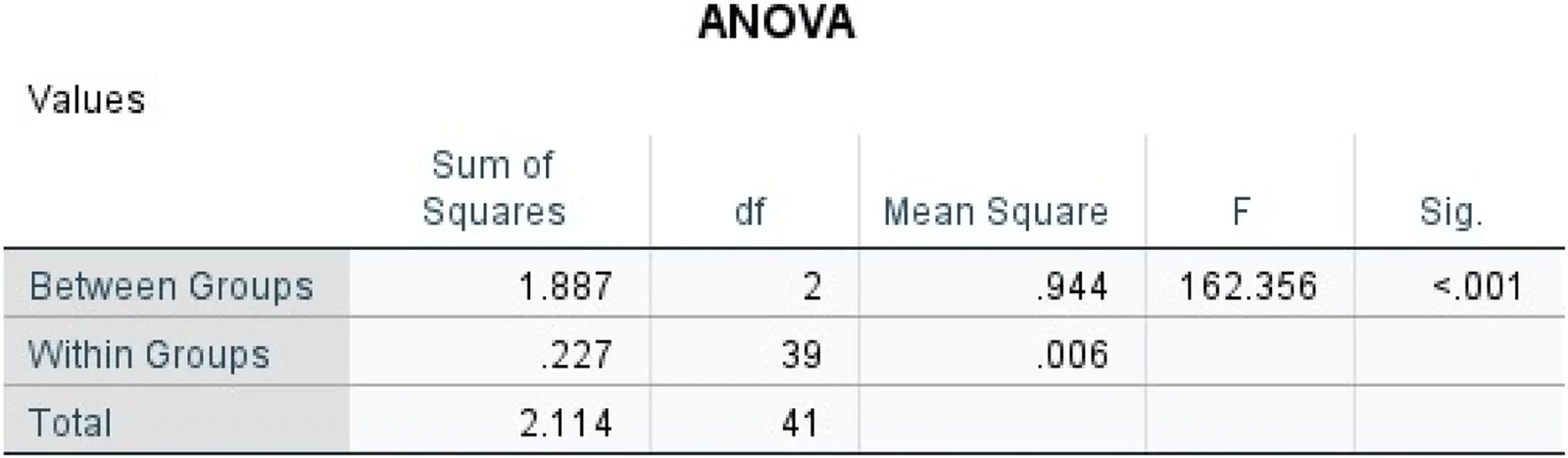
Shows a highly significant difference between the study groups (*p* < 0.001), with a large F-value of 162.356, indicating that the differences in mean values among the materials are statistically significant

**Table 4:**
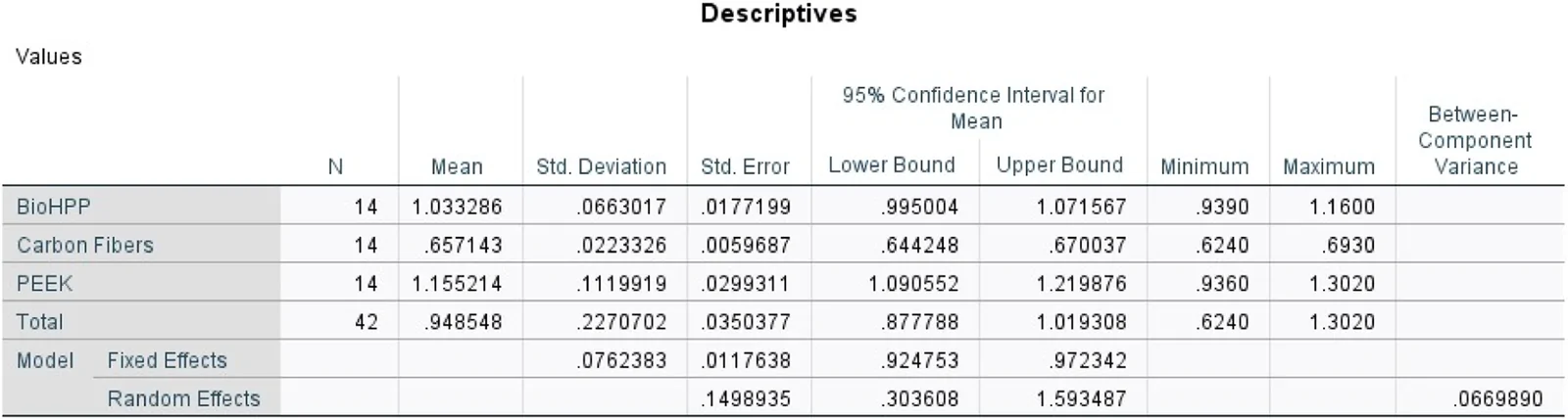
Shows the descriptive statistics indicate that PEEK has the highest mean value (1.1552), followed by BioHPP (1.0333), and nano-carbon HPP (0.6571)

### Evaluation of hardness

**Fig. 11:**
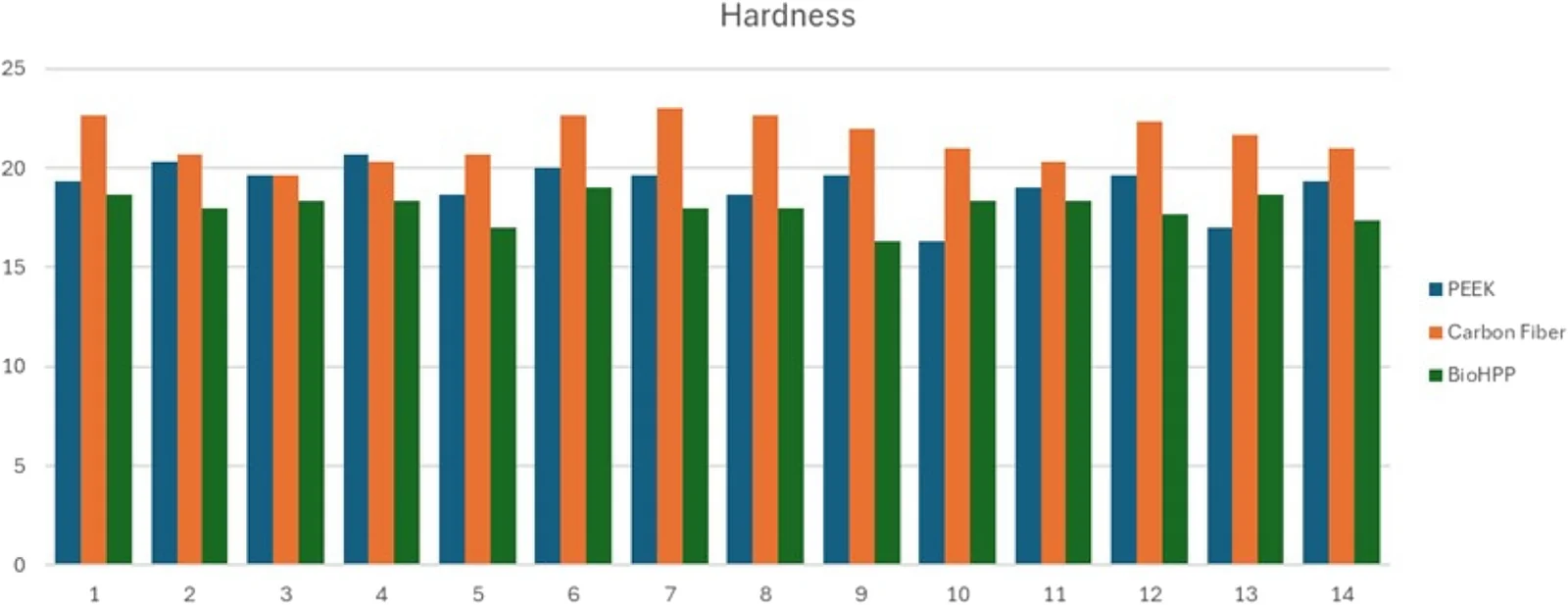
Shows a comparison between hardness values of three materials: PEEK, Nano-carbon HPP, and BioHPP in which Nano-carbon HPP consistently have the highest hardness values across all samples while BioHPP has the lowest hardness most of the time. PEEK is ioften slightly better than BioHPP, but clearly lower than Nano-carbon HPP

Table 5 shows the results of a Tukey HSD test comparing the means of three materials: BioHPP, Nano-carbon HPP, and PEEK. Significant differences were found between all material pairs (*p* < 0.05). Nano-carbon HPP had significantly higher values than both BioHPP and PEEK, while BioHPP also showed higher values than PEEK. The largest mean difference was between Nano-carbon HPP and BioHPP (3.643), indicating a strong distinction between these two materials. All confidence intervals exclude zero, confirming the statistical significance of the differences (Tables 6, 7 and Fig. 11).

**Table 5:**
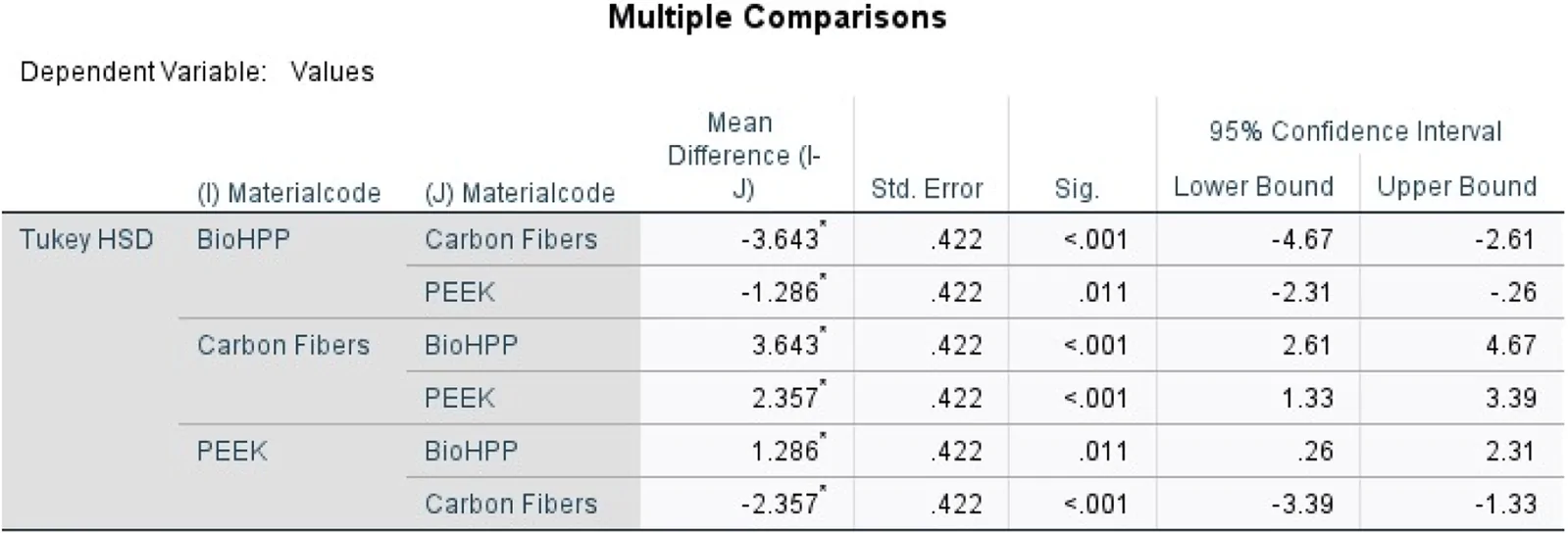
Shows the results of a Tukey HSD test comparing the means of three materials: BioHPP, Nano-carbon HPP, and PEEK

**Table 6:**
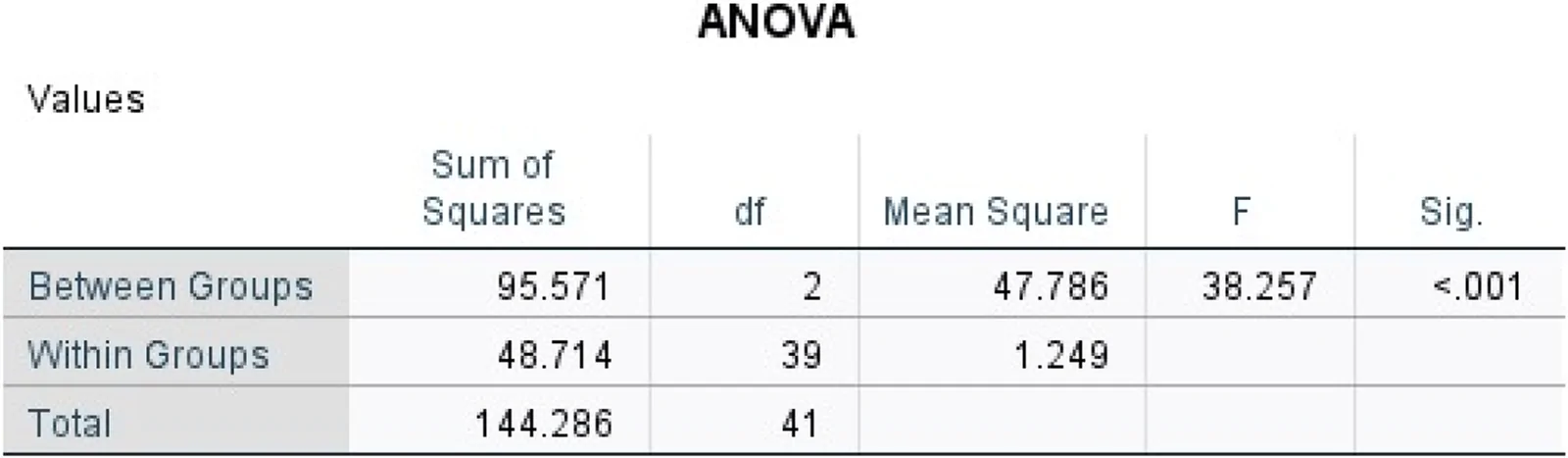
Shows the results of a one-way ANOVA used to test whether there are statistically significant differences between the means of three or more independent groups. Nano-carbon HPP has the highest mean, which likely indicates better performance

**Table 7:**
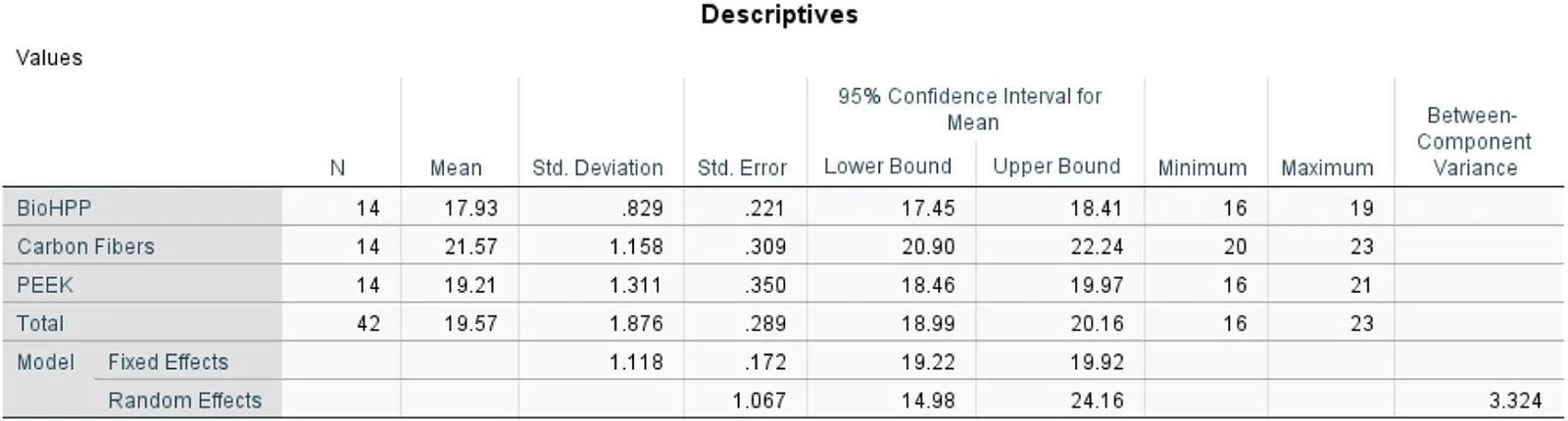
This table summarizes the descriptive statistics for each group. Nano-carbon HPP has the highest mean, and BioHPP the lowest value

## Discussion

The present in vitro study evaluated the surface roughness, hardness, and color stability of three non-metallic materials, BioHPP, PEEK, and nano-carbon reinforced polymers, used for implant-retained prosthetic applications. Statistically significant differences were observed among all tested materials, leading to rejection of the null hypothesis. These findings confirm that material composition and reinforcement strategy significantly influence the mechanical and surface behavior of polymer-based prosthetic materials used in implant dentistry [3]. 

Surface roughness is a clinically relevant factor due to its direct association with plaque accumulation, wear, and long-term prosthesis maintenance. In the current study, nano-carbon reinforced polymers exhibited the lowest surface roughness values, followed by BioHPP, while PEEK demonstrated the highest roughness. Menini et al. reported similar findings, demonstrating that carbon fiber–reinforced frameworks exhibit improved surface integrity and wear resistance compared with conventional polymer materials used in implant prosthodontics [3]. This finding may be attributed to the reinforcing effect of carbon fibers, which enhance structural homogeneity and resistance to surface degradation.

The higher surface roughness observed in PEEK specimens is consistent with previously published studies evaluating the behavior of PEEK under simulated oral conditions. Porojan et al. demonstrated that thermal cycling and water saturation significantly influence the surface characteristics of dental PEEK, resulting in increased roughness values over time. This behavior has been attributed to the semi-crystalline structure of PEEK and its susceptibility to hydrothermal aging, which may compromise surface smoothness in long-term clinical service [12]. 

Regarding hardness, nano-carbon reinforced polymers demonstrated significantly higher hardness values than both BioHPP and PEEK. This result aligns with the findings of Mahmood and Ibrahim, who reported that the incorporation of carbon nanotubes into polymer matrices significantly improves hardness and overall mechanical performance. The reinforcing effect of carbon-based fillers enhances load distribution within the material, increasing resistance to indentation and surface deformation [10, 11]. 

BioHPP exhibited intermediate hardness and surface roughness values compared with nano-carbon reinforced polymers and PEEK. As a ceramic-filled PEEK-based polymer, BioHPP combines favorable elasticity with improved mechanical performance. Previous studies have highlighted that BioHPP offers biomechanical advantages by reducing stress transmission to implants while maintaining sufficient rigidity for prosthetic frameworks, supporting its clinical application in implant-supported restorations [7–15]. 

Color stability is a critical esthetic parameter, particularly for prosthetic materials used in visible zones. In the present study, all tested materials exhibited varying degrees of discoloration following coffee immersion; however, nano-carbon reinforced polymers demonstrated superior color stability compared with PEEK and BioHPP. Similar observations were reported by Porojan et al., who found that PEEK-based materials are susceptible to color changes following aging and exposure to staining media, likely due to increased water sorption and pigment penetration [12]. 

From a clinical standpoint, materials exhibiting lower surface roughness, higher hardness, and improved color stability are expected to demonstrate enhanced resistance to wear, reduced plaque accumulation, and improved esthetic longevity. The superior performance of nano-carbon reinforced polymers observed in this study suggests their potential as a reliable metal-free alternative for implant-retained full-arch framework, particularly in patients with high functional demands and esthetic expectations. These findings are in agreement with recent literature emphasizing the growing role of high-performance polymers and fiber-reinforced composites in modern prosthodontics [7, 13]. 

Additionally, nanocarbon HPP can be used to fabricate the all superstructure of implant-supported prosthesis (framework and teeth). Unlike BioHPP and PEEK, where their use is limited only as framework and must be veneered with another more esthetic material with limitations and problems in bonding and surface treatment.

Despite the favorable outcomes observed, the limitations of this in vitro study must be acknowledged. Laboratory conditions cannot fully replicate the complex oral environment, including cyclic masticatory loads, pH variations, salivary enzymes, and microbial activity. In addition, this study evaluated only one thermocycling (aging) protocol, one staining solution, one polishing protocol, and one CAD/CAM manufacturing workflow; the findings may therefore not be generalizable to other aging regimens, staining media, surface-finishing techniques, or fabrication systems. Therefore, although the results are promising, further in vitro studies using additional aging and staining protocols and measuring the PH of the staining solution, together with in vivo investigations assessing mechanical properties, biocompatibility, and bonding behavior of nano-carbon reinforced polymers with dental adhesives and cements under simulated and clinical oral conditions, are required to validate the clinical performance and longevity of these materials in implant-supported prosthetic applications [16]. 

## Conclusion

Within the limitations of this in vitro study, nano-carbon reinforced polymer exhibited significantly lower surface roughness, higher hardness, and better color stability than BioHPP and PEEK after thermocycling and staining. These findings suggest that nano-carbon reinforced polymer may represent a promising metal-free alternative for implant-supported prosthetic frameworks; however, further in vitro studies using additional aging and staining protocols, as well as in vivo and long-term clinical investigations, are needed before firm clinical recommendations can be made.

## Data Availability

All datasets and materials used and/or analyzed during the current study are included in this published article.

## Abbreviations

PEEK: Poly-Ether-Ether-Ketone
BioHPP: Biocompatible high‑performance polymer
CAD/CAM: Computer-Aided-Design/Computer-Aided-Manufacturing
Ra: Surface roughness average
VHN: Vickers hardness number
ISO: International Organization for Standardization
